# Homogenization and Equivalent Beam Model for Fiber-Reinforced Tubular Profiles

**DOI:** 10.3390/ma13092069

**Published:** 2020-04-30

**Authors:** Daniel Gnoli, Sajjad Babamohammadi, Nicholas Fantuzzi

**Affiliations:** 1 DICAM Department, University of Bologna, 40136 Bologna, Italy; daniel.gnoli@studio.unibo.it; 2 Gruppo COSMI, 48122 Ravenna, Italy; sajjad.babamohammadi@gruppocosmi.com

**Keywords:** pultruded beams, effective stiffness matrix, FRP, hollow circular beams, finite element method

## Abstract

The current work presents a study on hollow cylinder composite beams, since hollow cylinder cross-sections are one of the principal geometry in many engineering fields. In particular, the present study considers the use of these profiles for scaffold design in offshore engineering. Composite beams cannot be treated as isotropic ones due to couplings mainly present among traction, torsion, bending and shear coefficients. This research aims to present a simple approach to study composite beams as they behave like isotropic ones by removing most complexities related to composite material design (e.g., avoid the use of 2D and 3D finite element modeling). The work aims to obtain the stiffness matrix of the equivalent beam through an analytical approach which is valid for most of the laminated composite configurations present in engineering applications. The 3D Euler–Bernoulli beam theory is considered for obtaining the correspondent isotropic elastic coefficients. The outcomes show that negligible errors occur for some equivalent composite configurations by allowing designers to continue using commercial finite element codes that implement the classical isotropic beam model.

## 1. Introduction

Hollow cylinders are one of the most common elements in offshore engineering, e.g., risers, pipes and generally used for scaffolding systems. Among vast materials to produce these kinds of elements, all of them have one mutual defect which is an important factor in an offshore environment; that is corrosion. The most common material used in offshore structures is steel, which although it is stiff and light (compared to reinforced concrete) but it can be easily corroded, which makes maintenance activities very expensive. On the other hand, plastics, a non-environmentally friendly material, can be in this context environmental-friendly. This is due to the fact that fewer materials are involved due to less frequent maintenance, thus, less resources. Steel structural elements should be changed frequently in an offshore environment to maintain offshore structural integrity. Steel is used also in onshore civil applications when being lightweight is an important prerequisite at the design phase. One of the plastics’ problem is the low stiffness and strength compared to steel. To overcome this problem fibers can be added in order to make the so-called Fiber Reinforced Plastics (FRPs). FRPs can be produced by different methods which leads to different stiffness and strength. However, composite materials do not behave like well-known isotropic ones. Therefore over the years, several methodologies have been utilized to analyze composite materials and structures simply and accurately.

Regarding hollow circular cross-sections, the stress distribution of anisotropic configurations has been led by Lekhnitskii [1] which was a starting point for further investigations by other scientists and engineers. Based on this, Jolicoer and Cardou [2] studied hollow cylinders under bending, tensile and torsions. The hollow cylinders behavior under hygrothermal and mechanical loads was investigated by Kollar et al. [3,4]. They followed displacement-based approach in their works, as well as Bhaskar and Varadan [5], Xia et al. [6,7], Calhoglu et al. [8] and Bakian et al. [9]. Verijenko et al. [10] studied the behavior of laminated cylinder under internal and external pressures. Roque and Ferreira [11] studied plates and shell deformation by means of Reddy’s theory. Salahifar and Mohareb [12] studied shell cylinders under harmonic forces. Laminated tubes were also considered by Tarn et al. [13] to present a state space approach to torsion, extension, bending, shearing and pressure. Bai et al. [14] investigated the buckling behavior of thermoplastic pipes under combined bending and tension loads. Derisi et al. [15] and Shadmehri et al. [16] studied the composite tubes under bending. Effect of pressure, shear and torsion on anisotropic materials was analyzed by Ting [17,18]. Dynamic axial compression on thin wall circular tubes was investigated by Uchikawa et al. [19]. Non-classical effect on hollow cylinders was studied by Silvestre [20] which led us to present a formula on generalized beam theory. Kardomateas [21] investigated orthotropic shells under internal and external pressure. Wang et al. [22] presented a study on compressive behaviors of FRP tubular beams. In addition, other works [23,24,25,26,27] have been presented on the investigation of different laminated composites subjected to several loadings. Different investigations have been done on multi-layered hollow cylinders [28,29]. Khalili et al. [30] analyzed the dynamic behavior of multilayered composite plates. Experimental data and laboratory tests were provided by Ascione et al. [31], Boscato and Russo [32], Philippidis and Vassilopoulos [33] and Quadrino et al. [34] to analyze the behavior of composite laminates. Ascione et al. [31] presented a bearing design formula, Boscato and Russo [32] presented dynamic parameters, Philippidis and Vassilopoulos [33] presented the effect of off-axis loading o fatigue and static behavior, whereas Quadrino et al. [34] investigated the local behavior of commercial I-shaped tubes. Furthermore, Madenci et al. [35] used both experimental and theoretical data to investigate the effect of flexure and Xin et al. [36] in-plane compression and shear on GFRPs. Experimental studies also have been done by Mayookh et al. [37] on the flexural creep of unidirectional bars and by Zhang et al. [38] on correlation between mechanical properties of FRPs. A homogeneous approach was considered by Sun and Li [39] to investigate the load-deformation of thick laminate and Sun et al. [40] to study the stress analysis of hollow cylinders. Other current applications of FRP tubular profiles have been presented in for retaining walls [41], column jacketing [42] and reinforcement of concrete slabs [43].

Homogenization is a method to investigate the macroscopic behavior of a material by considering a replacement of that material to an equivalent homogeneous one [44,45,46]. Thick hollow beams were investigated by Kim and White [47]. In addition, Yildiz and Sarikanat [48] used finite element analysis to investigate the material properties of multilayered hollow composites; whereas, Ferreira [49] used global meshless approximation to analyze them. Yazdani Sarvestani et al. [50] investigated the thick orthotropic cantilever under transverse loading and the effect of shear on stress distributions of thick composites [51]. Theory on governing equations of anisotropic thin-walled members was presented by Berdichevski et al. [52]. Kollar and Pluzsik [53] studied thin-walled composite beams and presented a theory to calculate the stiffness matrix of closed sections. Jung and Lee [54] investigated thin-walled I-beam composites. Lateral buckling of thin-walled composites was investigated by Lee [55]. Dynamic response of FRPs have been investigated by Boscato [56]. Corotational method was used by de Miranda et al. [57] and Ruggerini et al. [58] to investigate post-buckling and non-linear GBT buckling analysis, respectively.

Lately, Babamohammadi et al. [59] presented research based on a homogenization method of composite beams which lead to a simple design procedure of frame structures made of FRP beams. The present research develops further and expands such previous work by including an in-depth analysis of the mechanical behavior of hollow composite beams with several standards and not-standard lamination schemes as well as critical discussion and remarks on possible applications of the present methodology for practical engineering purposes.

## 2. Motivation

The mechanical behavior of isotropic beams is well-known in engineering practices and it is used for designing any kind of frame structure. However, if the beam is made up of fiber-reinforced composites its behavior cannot be attributed to one of the isotropic materials.

Stiffness matrices of hollow cylindrical composite structures can be determined by employing a finite element program. In this work, ABAQUS software [60] has been utilized for hollow profiles of 4 mm thickness which is the standard dimension of scaffolding beam components. Several stacking sequences are analyzed with different arrangements of the reinforcing fibers to investigate different coupling effects given by the composite configuration in comparison with the classical isotropic model.

The stiffness matrix of a two-node Euler–Bernoulli isotropic beam with compact cross-section, in the 3D space, has six degrees of freedom per node as 12×12 matrix $\left[K\right]=k_{i,j}$ for i,j=1,2,…,12. However, it is not necessary to define 144 parameters. If the cross-section is compact and with a double-symmetry (as in the present case) it is sufficient to compute six indipendent parameters. Below definitions of the stiffness matrix components are given.

(1) $$\left[K\right]=\left[\begin{array}{cccccccccccc} k_{1,1} & 0 & 0 & 0 & k_{1,5} & 0 & k_{1,7} & 0 & 0 & 0 & k_{1,11} & 0\\ \; & k_{2,2} & 0 & k_{2,4} & 0 & 0 & 0 & k_{2,8} & 0 & k_{2,10} & 0 & 0\\ \; & \; & k_{3,3} & 0 & 0 & 0 & 0 & 0 & k_{3,9} & 0 & 0 & 0\\ \; & \; & \; & k_{4,4} & 0 & 0 & 0 & k_{4,8} & 0 & k_{4,10} & 0 & 0\\ \; & \; & \; & \; & k_{5,5} & 0 & k_{5,7} & 0 & 0 & 0 & k_{5,11} & 0\\ \; & \; & \; & \; & \; & k_{6,6} & 0 & 0 & 0 & 0 & 0 & k_{6,12}\\ \; & \; & \; & \; & \; & \; & k_{7,7} & 0 & 0 & 0 & k_{7,11} & 0\\ \; & \; & \; & \; & \; & \; & \; & k_{8,8} & 0 & k_{8,10} & 0 & 0\\ \; & \; & \; & \; & \; & \; & \; & \; & k_{9,9} & 0 & 0 & 0\\ \; & \; & \; & \; & \; & \; & \; & \; & \; & k_{10,10} & 0 & 0\\ \; & \; & \; & \; & \; & \; & \; & \; & \; & \; & k_{11,11} & 0\\ \mathrm{sym} & \; & \; & \; & \; & \; & \; & \; & \; & \; & \; & k_{12,12} \end{array}\right]$$

that can be written in compact matrix form as

(2) $$\left[K\right]=\left[\begin{array}{cccc} \left[k_{1}\right] & \left[k_{2}\right] & -[k_{1}] & \left[k_{2}\right]\\ \; & \left[k_{3}\right] & \left[k_{2}\right] & \left[k_{4}\right]\\ \; & \; & \left[k_{1}\right] & -[k_{2}]\\ \mathrm{sym} & \; & \left[k_{3}\right] \end{array}\right]$$

where

(3) $$\begin{array}{c} \left[k_{1}\right]=\left[\begin{array}{ccc} \frac{12EI}{L^{3}} & 0 & 0\\ 0 & \frac{12EI}{L^{3}} & 0\\ 0 & 0 & \frac{EA}{L} \end{array}\right],\;\left[k_{2}\right]=\left[\begin{array}{ccc} 0 & \frac{6EI}{L^{2}} & 0\\ -\frac{6EI}{L^{2}} & 0 & 0\\ 0 & 0 & 0 \end{array}\right]\\ {}_{3}]=\left[\begin{array}{ccc} \frac{4EI}{L} & 0 & 0\\ 0 & \frac{4EI}{L} & 0\\ 0 & 0 & \frac{GJ}{L} \end{array}\right],\;\left[k_{4}\right]=\left[\begin{array}{ccc} \frac{2EI}{L} & 0 & 0\\ 0 & \frac{2EI}{L} & 0\\ 0 & 0 & -\frac{GJ}{L} \end{array}\right]. \end{array}$$

E,G are classical elastic properties and A,I,L are cross-section area, the moment of inertia and length of the beam. Definitions for the sitffness components $k_{i,j}$ for i,j=1,2,…,12 in Equation (1) are straightforwardly given by comparison with definitions in Equation (3). It is clear that for isotropic beams only six parameters are sufficient to define the whole stiffness matrix of the beam.

As far as structures made of composite materials are concerned, there are still no relations available that define the stiffness matrix within a specific theoretical framework. However, it can be demonstrated that orthotropic beams (with fibers parallel to the beam’s principal axis) have the same nonzero elements in the stiffness matrix as the equivalent isotropic one. Thus, if homogenized mechanical properties can be carried out for composites hollow beams the approach for isotropic beams can be transferred to composite configurations. In this regard, a study was carried out by Reddy [61] wherein the bending, vibration and buckling plate problems of laminated composite plates were compared by increasing the number of plies in cross- and angle-ply configurations. Reddy showed that by increasing number of laminae in cross-ply orientation, the mechanical behavior of the plate tends to be equivalent to the same plate in orthotropic configuration (e.g., single-ply with orientation 0). When angle-ply plates are studied, the mechanical characteristic is asymptotic to behavior between (45–45) and (45–45) s and does not change when the number of layers is large.

The latter has been generalized to hollow circular beams in order to see if such asymptotic behavior could be used to simply model these beams as equivalent isotropic or orthotropic ones.

## 3. Theoretical Background

Taking the studies of Reddy [61] on laminated composite plates as a reference, in the following, sections cross- and angle-ply laminated hollow circular beams are investigated. The geometric and mechanical properties considered are listed in Table 1. Note that geometric properties selected are the ones typical of beams used in scaffolding systems. Optimization of cross-section properties is not analyzed because out of the scope of the present work. Mechanical properties taken here are typical engineering constants of Carbon FRP (CFRP). This study is not limited to this geometry and/or mechanical properties but aims to present a generalized framework that is able to work in every context.

The stiffness matrix of the composite beam has been computed using ABAQUS as described in [59] using a 3D shell model and laminated shell elements of first-order (Mindlin theory). The cylindrical geometry is restrained at the two ends through rigid links which simulate the two end nodes of the equivalent beam. Stiffness matrix components (following the structure given by Equation (2)) are determined by setting alternatively one unitary displacement and by retrieving the corresponding boundary forces. For each unitary displacement, a stiffness matrix row is carried out. Since the equivalent beam has two nodes with 6 degrees of freedom each, the stiffness matrix result to be 12×12. In addition, equivalent elastic properties via an analytical cross-section homogenization by Sun et al. [40] is also considered. The present methodology is based on the ensemble of the latter. Interested readers are asked to refer to the cited works for further details on such procedures that are not reported below for the sake of conciseness.

### 3.1. Cross-Ply Laminates

**Figure 1:**
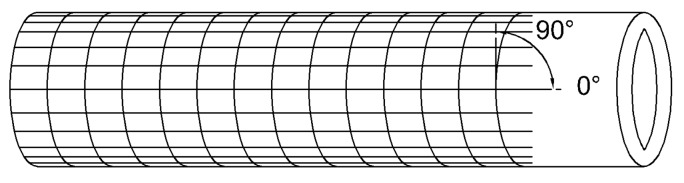
Cross-ply laminates.

**Figure 2:**
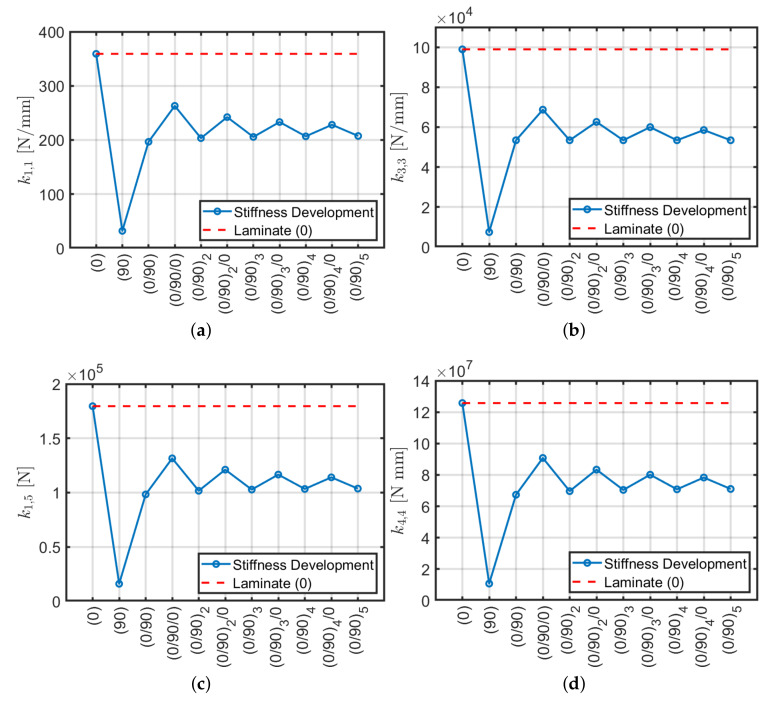

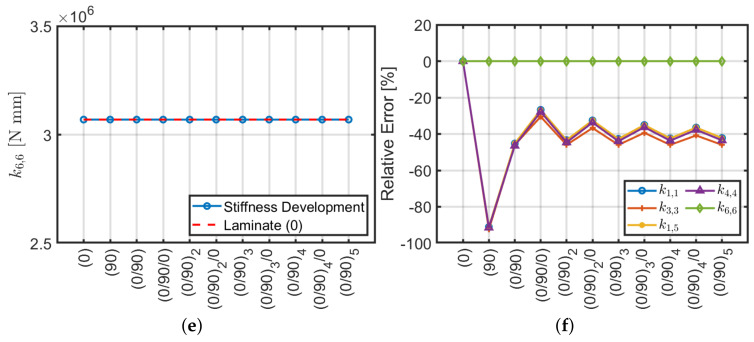
Stiffness trends with respect to the number of plies (**a**–**e**) and relative error with respect to an orthotropic conﬁguration (**f**).

A typical circular hollow profile with cross-ply configuration is depicted in Figure 1. The stiffness matrix of composite beams with a different number of plies is presented below with geometric and mechanical properties listed in Table 1. Configurations are alternatively symmetric and antisymmetric (with the only exception of the first two which are (0) and (90)). All the nonzero stiffness matrix components are compared with the same given by an orthotropic configuration (0). Figure 2a–e display such comparison and a summary in terms of relative error is depicted in Figure 2f where the error tends to be −40% by increasing the number of plies.

Clearly, the global trend of all stiffness constants deviates from the initial orthotropic behavior and becomes stable when 3 or 4 layers are considered. Thus, it can be established that the study of Reddy [61] for cross-ply plates does not apply to hollow circular cylinders but it is closer to the behavior of the angle-ply plates. In detail, it can be noted that $k_{1,1}$, $k_{3,3}$, $k_{1,5}$ and $k_{4,4}$, decrease until reaching an almost constant value. Specifically, symmetrical configurations reach this value from above, whereas the others from the bottom. The variation in torsional stiffness $k_{6,6}$ is negligible since it does not deviate from the initial configuration, thus torsional stiffness does not improve by introducing more cross-ply layers. In conclusion, it is possible to confirm that by increasing the number of layers in a cross-ply lamination scheme, keeping a constant thickness, about 40% loss of stiffness is generated, while the torsional stiffness remains almost constant.

**Figure 3:**
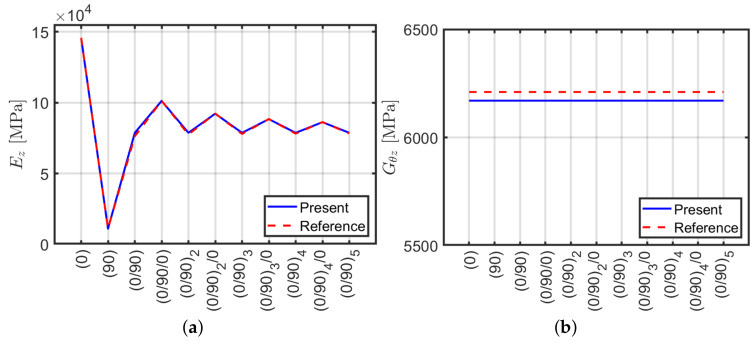
Equivalent moduli using ABAQUS and Sun et al. [40] approach: (**a**) normal $E_{z}$; (**b**) shear $G_{\theta z}$.

The validity of the finite element model is tested by comparing it with the results of the analytical approach by Sun et al. [40] which provides homogenized modulus of elasticity $E_{z}$ and tangential modulus $G_{\theta z}$ for a composite cross-section in planar strain. For instance, by purging the stiffnesses $k_{3,3}$ (axial stiffness) and $k_{6,6}$ (torsional stiffness) computed with ABAQUS by L/A and L/J an equivalent elastic and shear moduli can be retrieved. It is recalled that $k_{3,3}=EA/L$ and $k_{6,6}=GJ/L$ for isotropic Euler–Bernoulli beam with compact cross-section as shown in Equation (3). The values obtained by both methods are shown in Figure 3 where they almost coincide. This demonstrates that ABAQUS and Sun et al. [40] method give the same results for the axial and torsional stiffnesses $k_{3,3}$ and $k_{6,6}$. Even though the two models have a different mathematical background they provide the same result because axial and torsional stiffnesses do not depend on beam slenderness as shear and bending behaviors do. ABAQUS model is introduced because Sun et al. [40] method does not carry out shear stiffness and it is not able to predict accurately bending behavior of composite beams [59]. Therefore one of the aims of the present work is to reply to this problem.

For the sake of conciseness, variation with respect to different mechanical or geometric properties is not reported. However, it has been observed by the authors that by varying the mechanical properties or geometric ratios (e.g., $E_{1}$, *A* and *L*) the results presented below are not affected (more details can be found in [62]). Thus, the data given represent a general FRP hollow beam and this study can be easily adapted to different materials or geometry.

### 3.2. Angle-Ply Laminates

**Figure 4:**
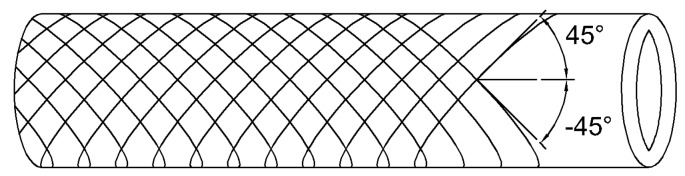
Angle-ply laminates.

**Figure 5:**
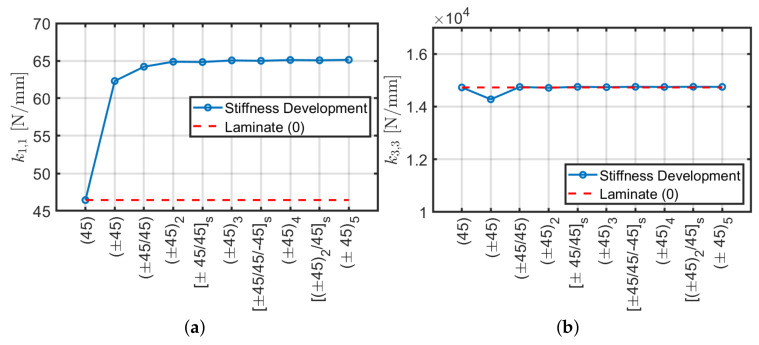

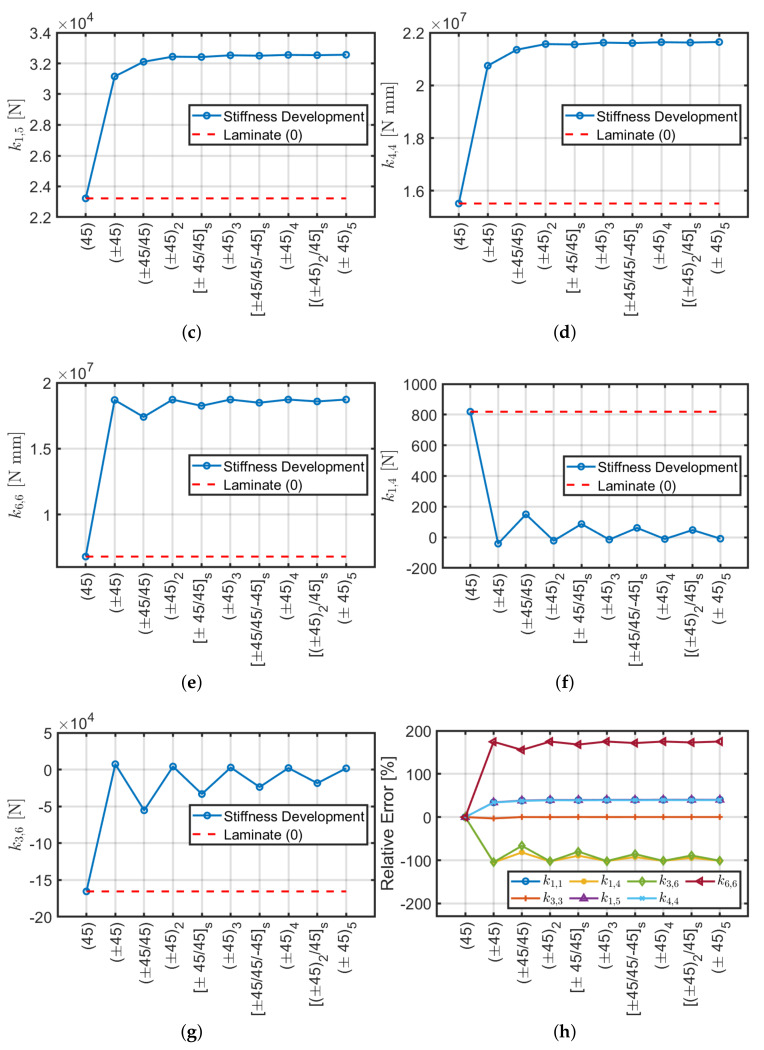
Stiffness trends with respect to number of plies (**a**–**g**) and relative error with respect to orthotropic conﬁguration (**h**).

Angle-ply lamination (±45/…) is used for structures that have to resist mainly against torsion or internal pressure. A typical configuration is depicted in Figure 4. The same geometric properties of the previous case are considered in the following and the number of plies is increased starting from the single-ply (45). Trends of the stiffness values for each configuration are assessed. Considering a constant thickness of 4 mm, Figure 5 shows the plots of the stiffness matrix coefficients and the relative error with respect to the scheme of initial lamination (45).

By examining the graphs obtained (Figure 5) it is possible to remark that compared to the cross-ply configuration, the stiffnesses $k_{1,1}$, $k_{1,5}$ and $k_{4,4}$, tend to increase to a constant value regardless of the lamination scheme, whether symmetrical or not. The axial stiffness $k_{3,3}$ approximately keeps a constant value. For the torsional stiffness $k_{6,6}$, a substantial increase is noted. Since the angle of the reinforcing fibers differs from the longitudinal (0) and transverse (90) directions, coupling stiffnesses appear due to shear-bending ($k_{1,4}$) and axial-torsional ($k_{3,6}$). This agrees with Reddy’s studies on angle-ply reinforced plates: the structural strength has an asymptotic behavior by increasing the number of plies in the stacking sequence. In conclusion, with reference to Figure 5h it is possible to observe a variation of about 40% of the stiffness values $k_{1,1}$, $k_{1,5}$ and $k_{4,4}$ analogous to the previous cross-ply case, whereas $k_{3,3}$ has approximately a constant trend, practically is characterized by zero relative error. It is remarked that coupling stiffnesses $k_{1,4}$ and $k_{3,6}$ tend to zero by increasing the number of plies (100% of relative error with respect to (45) configuration). Thus, for these stacking sequences, the composite tends to have an orthotropic behavior with very small couplings.

**Figure 6:**
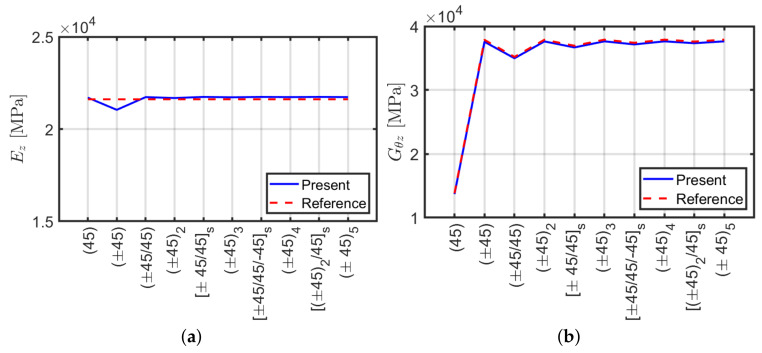
Equivalent moduli using ABAQUS and Sun et al. [40] approach: (**a**) normal $E_{z}$; (**b**) shear $G_{\theta z}$.

Analogously to the previous section a comparison between ABAQUS and an analytical formulation [40] is performed for angle-ply configurations. Stiffness terms $k_{3,3}$ and $k_{6,6}$ are carried out via ABAQUS and purged by multiplication of L/A and L/J, respectively in order to derive $E_{z}$ and $G_{\theta z}$. Such comparison is represented in Figure 6. It can be observed that both methods give the same results in terms of $k_{3,3}$ and $k_{6,6}$ as in the cross-ply case presented in the previous section.

In conclusion, the present ABAQUS model is able to approximate accurately the homogenized normal $E_{z}$ and shear $G_{\theta z}$ moduli by considering axial $k_{3,3}$ and torsional $k_{6,6}$ stiffness components, respectively.

## 4. Beam Slenderness Effect

As aforementioned, shear and bending stiffness depend on beam slenderness and they are not predicted accurately by Sun et al. [40] method. Therefore, the aim of the present section is to show such behaviors for several laminates. The geometry of the cross-section is kept constant and beam length is increased from 1 to 5 m. For cross-(0/90/…) and angle-ply (±45/…) laminations the number of plies is increased as in the previous section. Moreover, quasi-isotropic configuration (0/±45/90)${}_{s}$ is taken into consideration as well as other particular stacking sequences taken from the literature. Main stiffness components are represented in a dimensionless form with respect to the correspondent stiffness component given by the Euler–Bernoulli theory. For instance shear stiffness $k_{1,1}$ is multiplied by $L^{3}/\left(12E_{z}I\right)$, bending stiffness $k_{4,4}$ by $L/(4E_{z}I)$, shear-bending stiffness $k_{1,5}$ by $L^{2}/\left(6E_{z}I\right)$, axial stiffness $k_{3,3}$ by $L/E_{z}A$ and torsional stiffness $k_{6,6}$ by $L/(G_{\theta z}J)$. In addition, the extra shear-bending term $k_{4,11}$ which is not classical is compared to its main stiffness contribution on the main diagonal $k_{4,4}$. The homogenized moduli $E_{z}$ and $G_{\theta z}$ are computed by inverting axial and torsional stiffnesses, respectively, as shown in the previous section.

### 4.1. Cross-Ply Laminates

**Figure 7:**
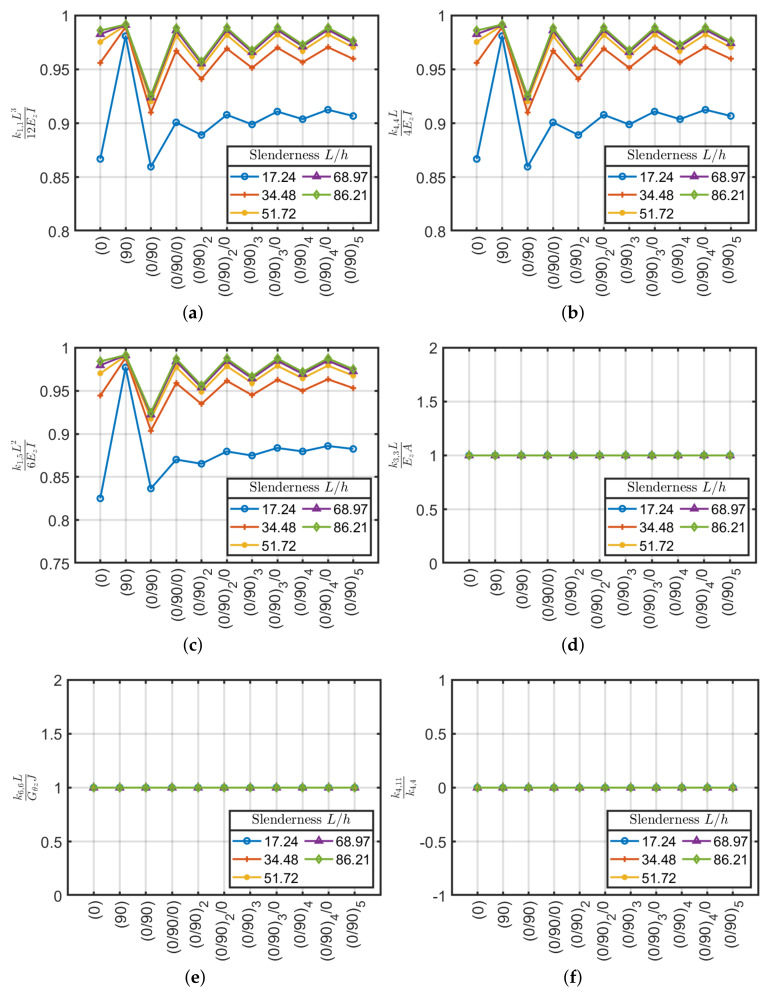
Stiffness coefficients (**a**) $\frac{k_{1,1}L^{3}}{12E_{z}I}$, (**b**) $\frac{k_{4,4}L}{4E_{z}I}$, (**c**) $\frac{k_{1,5}L^{2}}{6E_{z}I}$, (**d**) $\frac{k_{3,3}L}{E_{z}A}$, (**e**) $\frac{k_{6,6}L}{G_{\theta z}J}$ and (**f**) $k_{4,11}/k_{4,4}$ as a function of slenderness L/h for cross-ply laminates.

Results obtained are depicted in Figure 7 where, as expected, axial $k_{3,3}$ and torsional $k_{6,6}$ rigidity do not depend on beam slenderness. In addition, as already expected no bending coupling is observed, thus $k_{4,11}=0$. Moreover, the $E_{z}$ and $G_{\theta z}$ values coincide with the ones given by Sun et al. [40] formulation. On the contrary shear $k_{1,1}$, bending $k_{4,4}$ and shear-bending $k_{1,5}$ moduli tend to follow classical formulas (e.g., $k_{1,1}=12E_{z}I/L^{3}$, $k_{4,4}=4E_{z}I/L$) only for large slenderness. In particular, there is a more accelerated trend for symmetrical configurations.

It should be remarked that the calculation of the stiffness matrix by using classical formulas varies with beam slenderness. This error leads to lower stiffness and consequently, it should be considered in the design phase by the introduction of a correction factor. For symmetric lamination schemes and with slenderness values L/h>30–35 the error is less than 5%.

### 4.2. Angle-Ply Laminates

**Figure 8:**
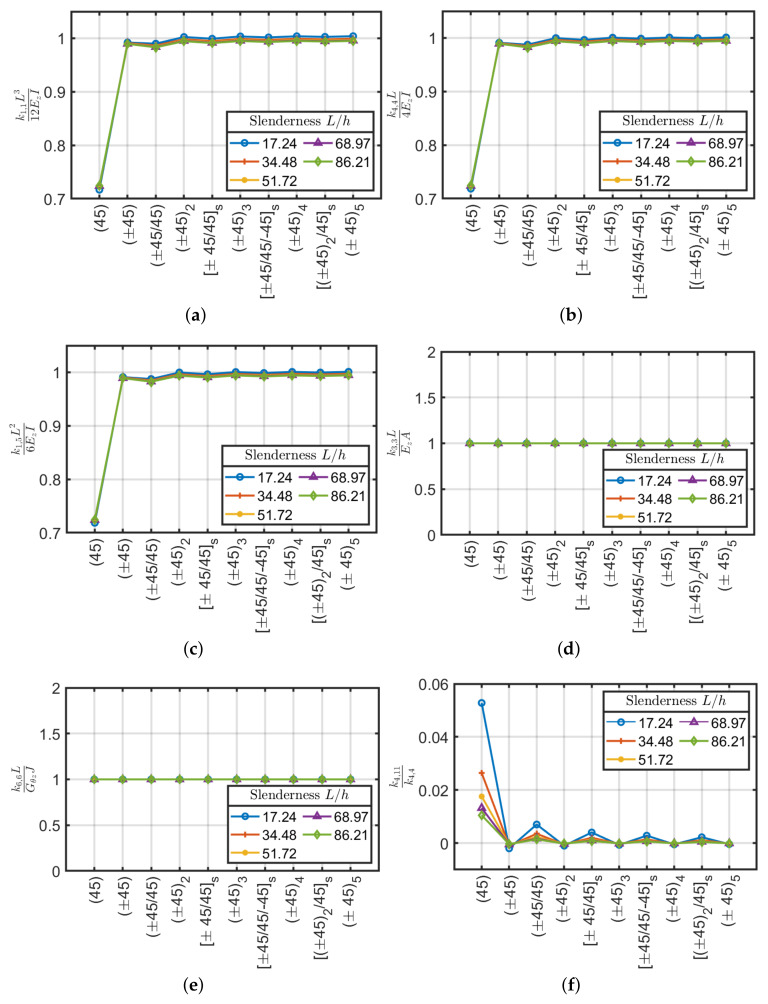
Stiffness coefficients (**a**) $\frac{k_{1,1}L^{3}}{12E_{z}I}$, (**b**) $\frac{k_{4,4}L}{4E_{z}I}$, (**c**) $\frac{k_{1,5}L^{2}}{6E_{z}I}$, (**d**) $\frac{k_{3,3}L}{E_{z}A}$, (**e**) $\frac{k_{6,6}L}{G_{\theta z}J}$ and (**f**) $k_{4,11}/k_{4,4}$ as a function of slenderness L/h for angle-ply laminates.

For the angle-ply configurations (±45/...) the values compared between the stiffnesses generated by ABAQUS and the Euler–Bernoulli theory for isotropic materials were depicted in Figure 8. For the present configuration, as in the previous cross-ply one, the axial $k_{3,3}$ and torsional $k_{6,6}$ stiffness does not depend on the slenderness and the equivalent stiffness carried out are close to the ones obtained via Sun et al. [40] approach.

By considering shear and bending stiffnesses $k_{1,1}$, $k_{4,4}$ and $k_{1,5}$ the difference between the (45) laminate and the others can be easily noted. The single-ply configuration has approximately 30% of error with respect to classical formulas. By increasing the number of plies, on the contrary, the computed values are in agreement with the classical values if they were made of an equivalent isotropic material where the average error is below 2%. Antisymmetric lamination schemes have better behavior by excluding the scheme (±45) which shows a slightly higher stiffness (about 1%) than the reference ones. It is remarked that the angle-ply (±45/…) configuration has a very small variation with respect to beam slenderness. On the contrary, a large variation was observed for the cross-ply cases.

Finally, the angle-ply configuration (±45/…) has an extra coupling between bending moments ($k_{4,11}$) that is not present in the cross-ply configurations. This coupling tends to decrease by increasing the beam slenderness. Compared to the main bending stiffness $k_{4,4}$, this is minimal and can be considered negligible, except for the first lamination scheme (45) which has a difference of about 5%, consequently, they can be neglected for design purposes. The scheme (45) is feasible but not used in applications as it generates delamination and durability problems of the composite. Usually, the laminations are composed of several crossed layers, it is therefore legitimate, given the proposed results, to neglect the component $k_{4,11}$ in practical applications.

### 4.3. Quasi-Isotropic Configuration

**Figure 9:**
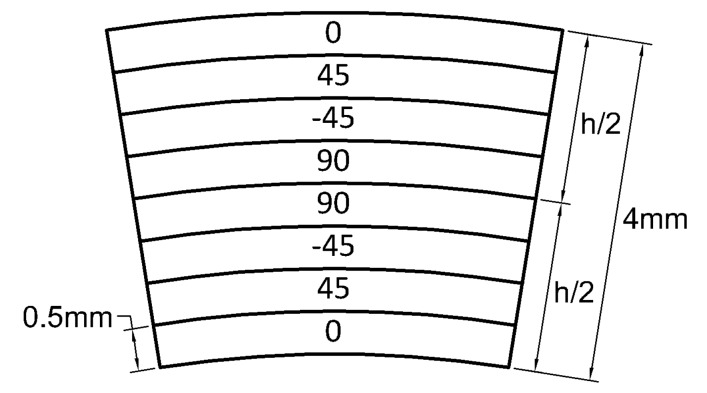
Quasi-isotropic laminate.

Quasi-isotropic laminates are made of three or more orthotropic plies of identical thickness and material. The stack considers for each ply +θ a corresponding one with −θ over the middle surface arranged with the same sequence order as depicted in Figure 9. In order to obtain an element with good shear, bending, traction and torsion properties a quasi-isotropic configuration with a combination of cross-(0/90/…) and angle-ply (±45/…) has been considered as (0/±45/90)${}_{s}$. The main feature of this typology is that the coupling stiffnesses $k_{1,4}$ and $k_{3,6}$ are zero. These laminates are widely used in practical applications, because they offer homogeneous stiffness and strength in all directions, and also have a good performance against crack propagation.

Stiffness components are carried out for various beam lengths considering the same geometric section and mechanical properties of the previous stacking sequences. The variations of the stiffness values with respect to the classical stiffnesses calculated as if an equivalent isotropic material with $E=E_{z}$ is considered. $E_{z}$ is calculated with Sun’s approach or by inverting the main component of axial stiffness $k_{3,3}$ given by the stiffness matrix by the ABAQUS model.

It is also observed that this configuration has values close to the calculated stiffness as if it was a homogenized isotropic material. As a matter of fact, stiffnesses $k_{1,1}$, $k_{4,4}$ and $k_{1,5}$ are all below 3% compared to the reference values. Furthermore, as the slenderness increases, this error decreases until it reaches a constant value of less than 1%.

The homogenized elastic $E_{z}$ and shear $G_{\theta z}$ moduli are obtained from axial $k_{3,3}$ and torsional $k_{6,6}$ stiffnesses by multiplying them by A/L and J/L, respectively. Thus, the ratios between $k_{3,3}$ and $k_{6,6}$ and the same calculated via Sun et al. [40] procedure are the same.

**Figure 10:**
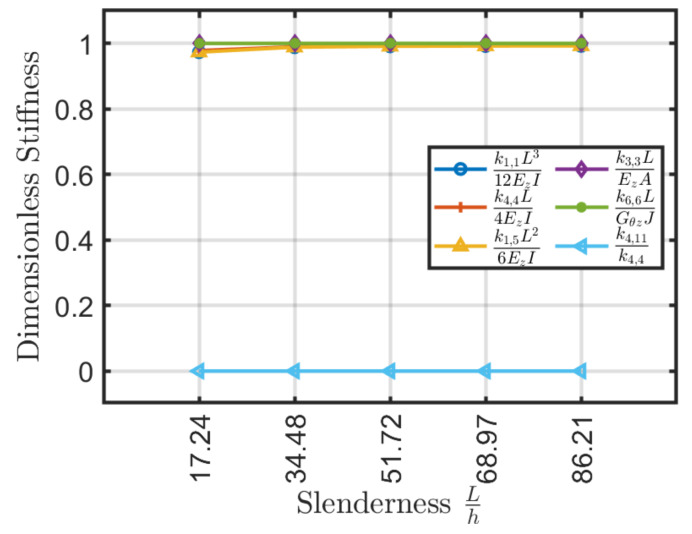
Stiffness coefficients $\frac{k_{1,1}L^{3}}{12E_{z}I}$, $\frac{k_{4,4}L}{4E_{z}I}$, $\frac{k_{1,5}L^{2}}{6E_{z}I}$, $\frac{k_{3,3}L}{E_{z}A}$, $\frac{k_{6,6}L}{G_{\theta z}J}$ and $k_{4,11}/k_{4,4}$ as a function of slenderness L/h for quasi-isotropic laminate.

It has been shown also for the quasi-isotropic configuration (0/±45/90)${}_{s}$, that stiffness ratios are almost constant with respect to the beam slenderness. In the current configuration, the stiffness due to the coupling between the moments ($k_{4,11}$) tends to increase until it reaches a constant value as the element’s slenderness increases; the values generated with respect to the bending stiffness ($k_{4,4}$) are, however, minimal and negligible (see Figure 10).

### 4.4. Bouligand Laminates

In this section, analyses are carried out by using bio-inspired Bouligand lay-ups of $5^{\circ }$ at each subsequent layer as presented by Mencattelli and Pinho [63] on laminated plates with ultra-thin thickness. It has been shown that by reducing the variation of the angles between the layers (e.g., the pitch Δθ) to the values that imitate the natural microstructure, it is possible to obtain at the same time a strong increase in the energy dissipated and in the maximum load-bearing capacity.

Bouligand unit considers rotation of 180${}^{\circ }$ inside each unit, however, 1/4 of a Bouligand unit has been considered here as the main unit. Thus, the following configurations and nomenclature will be used.

- $L.1\equiv {\left(0/5/\ldots /40/45\right)}_{s}$
- $L.2\equiv {\left(0/5/\ldots /85/90\right)}_{s}$
- $L.3\equiv {\left(0/5/\ldots /85/90/-85/\ldots /-5/0\right)}_{4}$
- $L.4\equiv {\left(0/5/\ldots /85/90/-85/\ldots /-5/0\right)}_{2s}$

**Figure 11:**
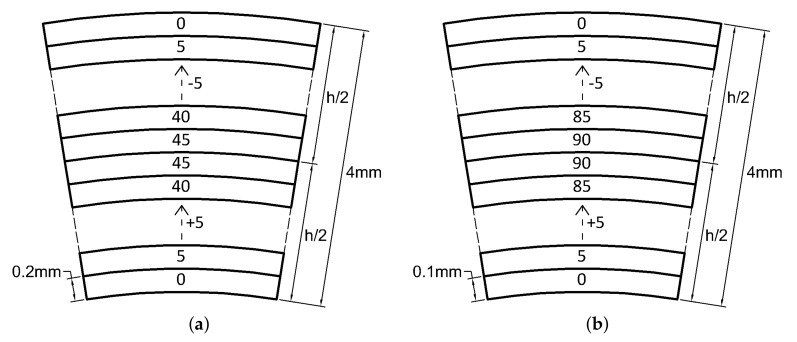

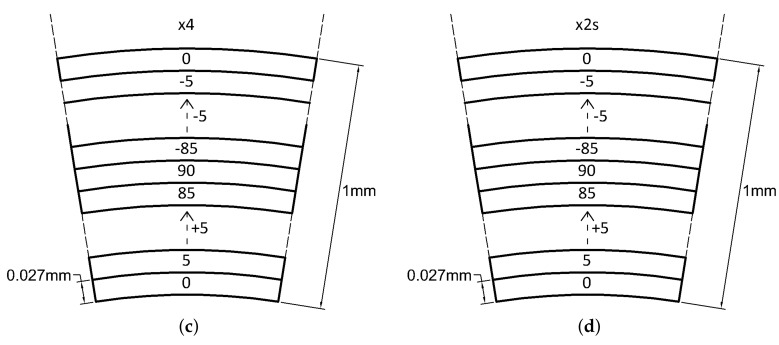
(**a**) *L*.1 laminate, (**b**) *L*.2 laminate (**c**) *L*.3 laminate (**d**), *L*.4 laminate.

A graphical representation of the four sequences considered are given in Figure 11. L.1 and L.2 are 1/4 and 1/2 of a Bouligand unit, respectively. L.3 and L.4 are an antisymmetric and a symmetry scheme inspired by Mencattelli and Pinho [63], respectively.

By keeping the same geometric section and mechanical properties of the previous cases, the following analyses are carried out on element lengths from 1 to 5 m. In order to correctly isolate the effect of the pitch angle of the reinforcement fibers Δθ, Mencattelli and Pinho [63] have decided to eliminate the couplings in the stiffness matrix by creating symmetric laminates. Therefore, L.4 adopts a symmetrical lamination scheme consisting of 2 symmetric modules (each module is defined by a complete set of the fiber angles, or $360^{\circ }$) with 146 layers in total:

(4) $$\begin{array}{c} {\left(0/5/\ldots /85/90/-85/\ldots /-5/0/5/\ldots /85/90/-85/\ldots /-5/0\right)}_{s}=\\ ={\left(0/5/\ldots /85/90/-85/\ldots /-5/0\right)}_{2s} \end{array}$$

**Figure 12:**
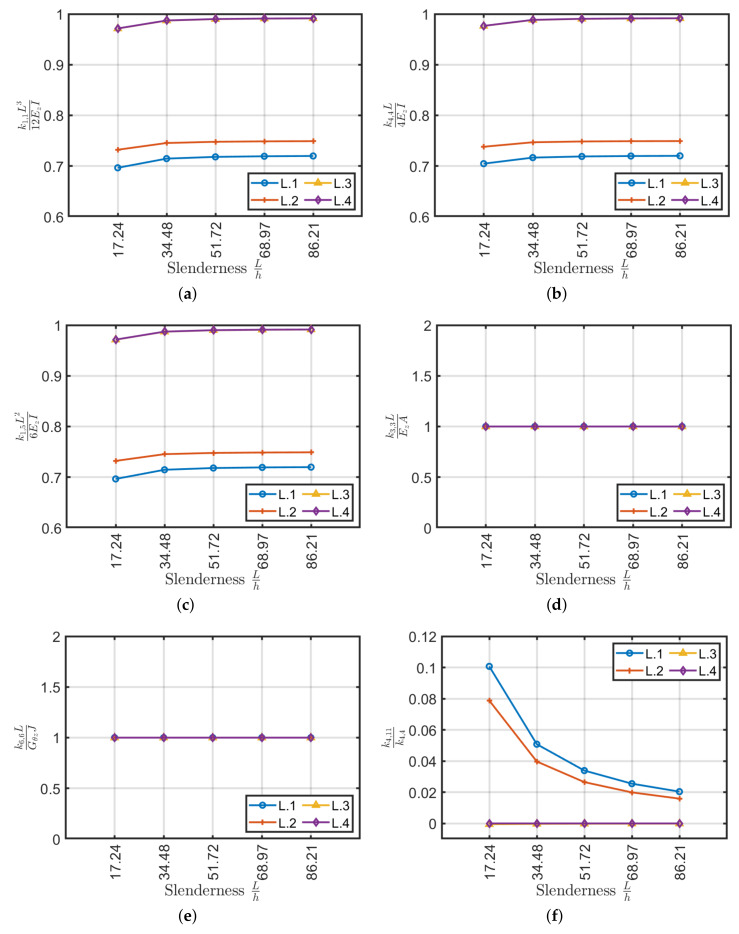
Stiffness coefficients as a function of slenderness for L.1, L.2, L.3 and L.4 (**a**) $\frac{k_{1,1}L^{3}}{12E_{z}I}$, (**b**) $\frac{k_{4,4}L}{4E_{z}I}$, (**c**) $\frac{k_{1,5}L^{2}}{6E_{z}I}$, (**d**) $\frac{k_{3,3}L}{E_{z}A}$, (**e**) $\frac{k_{6,6}L}{G_{\theta z}J}$ and stiffness ratio (**f**) $k_{4,11}/k_{4,4}$ for Bouligand laminates.

Stiffness variation as a function of the slenderness with respect to the classical stiffness relations are depicted in Figure 12. By using a symmetrical lamination scheme, in addition to the cancellation of the coupling stiffnesses, the variation between the stiffnesses $k_{1,1}$, $k_{4,4}$, $k_{1,5}$, $k_{3,3}$ and $k_{6,6}$ is further reduced with respect to the classical definitions. Likewise in this case, the relationship between the coupling stiffness $k_{4,11}$ and the bending stiffness $k_{4,4}$ is negligible.

**Figure 13:**
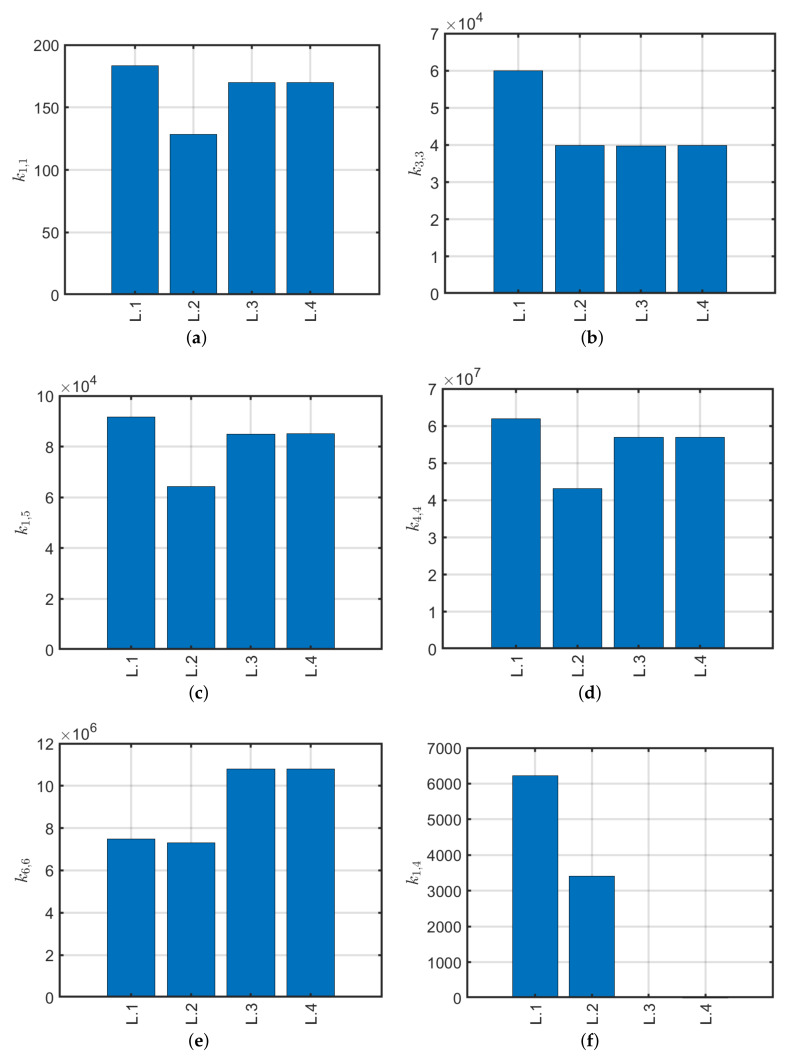

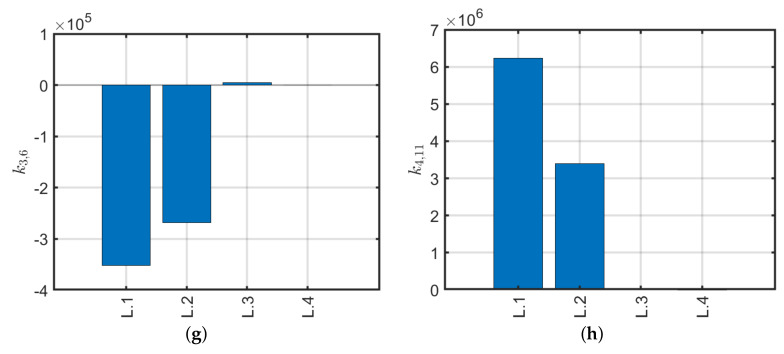
Stiffness components (**a**–**h**) of the considered Bouligand laminates for *L* = 1 m.

By comparing the obtained results, it is shown that the substantial differences between the selected lamination schemes are caused by the coupling stiffnesses $k_{1,4}$, $k_{3,6}$ and $k_{4,11}$. These coupling terms have higher values for laminates with few layers, even if symmetrical, and will tend to fade out by increasing the number of layers, thus tend to a balanced configuration. Although the first two configurations (L.1, L.2) are symmetrical, fairly pronounced coupling stiffnesses are generated. The validation of this behavior has been done through the Sun [40] model according to which coupling values are generated which are also different from zero and high between tension and torsion. Regarding Figure 13, it is possible to note that the laminate L.4 studied by Mencattelli and Pinho [63] has average axial, shear and bending stiffness values, high torsional and zero coupling stiffness values with respect to the others. For this reason, L.4 has been selected for the following comparisons with respect to the others presented.

## 5. Comparison

Following the analyses carried out, an overall view is given on the behavior of the various lamination schemes, evaluating the variation of the stiffnesses of the laminates with respect to the orthotropic scheme (0) and the coupling stiffnesses based on their relevance with respect to the correspondent main stiffness on the main diagonal of the stiffness matrix.

**Figure 14:**
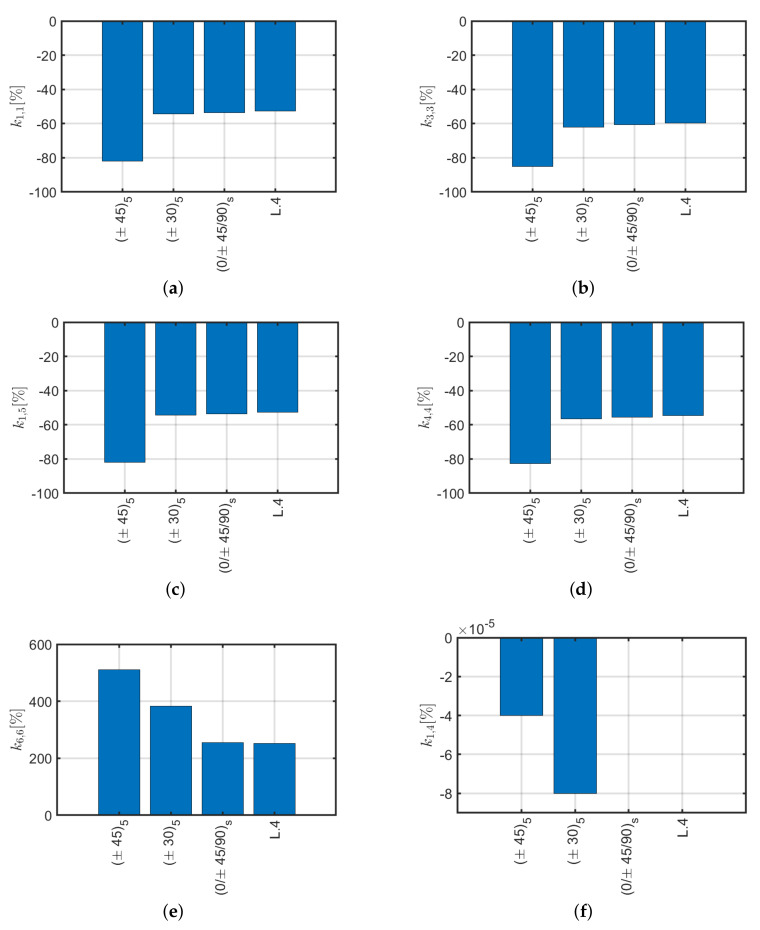

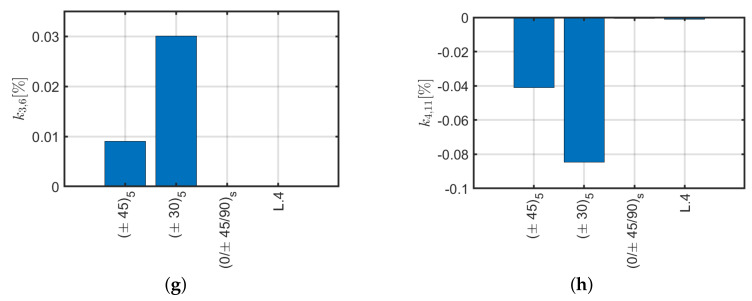
Comparison among stiffness components (**a**–**h**) for angle-ply, quasi-isotropic and L.4 *L*.4 laminates with *L* = 1 m respect to the orthotropic scheme (0).

Figure 14 shows the comparison for the main stiffnesses involved in the present study with respect to the reference (0) configuration. Note that angle-ply configurations have the highest stiffnesses compared to the others, but they also have shear-bending coupling $k_{1,4},k_{4,11}\neq 0$ and traction-torsion coupling $k_{3,6}\neq 0$. Whereas, quasi-isotropic and L.4 configurations show negligible couplings and lower stiffness values analogous to angle-ply configurations. From the obtained results the following comments arise:

- All the stiffnesses tends to have a constant value by increasing beam slenderness.
- The orthotropic scheme (0) is very efficient if subjected to shear, axial and bending actions, but not for torsional ones. However, it has a significant variation while increasing slenderness. Furthermore, it suffers from delamination, because the fibers being all parallel, tend to form a preferential fracture plane.
- The angle-ply scheme ${\left(\pm 45\right)}_{5}$ works conversely with respect to the orthotropic configuration (0). It has high torsional stiffness, but low axial, shear and bending ones. For the present configuration, the shear-bending coupling is negligible. It is possible to use Sun et al. [40] formulae to homogenize equivalent properties because its properties do not depend on the beam slenderness.
- The angle-ply scheme ${\left(\pm 30\right)}_{5}$ represents an intermediate solution between the orthotropic (0) and the angle-ply ${\left(\pm 45\right)}_{5}$ configurations. It has a lower axial, shear and bending stiffness of 50–60% approximately and torsional stiffness of 80% with respect to (0). The present stack, as well as ${\left(\pm 45\right)}_{5}$, has very low shear-bending coupling stiffness values which can be neglected and invariability of stiffness constants with respect to slenderness.
- The quasi-isotropic laminate (0/${\pm 45/90)}_{s}$ has stiffness values similar to the laminate ${\left(\pm 30\right)}_{5}$, except for the torsional stiffness which decreases by approximately 30%. Being a quasi-isotropic laminate, it has a negligible shear-bending coupling stiffness. In this case, the constants of the stiffness matrix vary slightly with increasing slenderness.
- The L.4 laminate, studied by Mencattelli and Pinho [63], with the only exception of the orthotropic configuration (0), presents slightly higher values for axial, shear and bending stiffnesses, but lower values for torsional stiffness with respect to the others. Coupling effects are minimal, and a very small dependency on the beam slenderness for the main stiffness components $k_{1,1}$, $k_{4,4}$, $k_{1,5}$, $k_{3,3}$ and $k_{6,6}$ is observed.

In conclusion, the best axial, shear and bending properties are achieved by the configuration (0) however such selection has very small torsional stiffness and low durability and reliability. Therefore, quasi-isotropic and L.4 configurations are suggested for having good mechanical performances in almost all conditions. These configurations do not present coupling terms so they can be easily characterized.

## 6. Laminates with Highly Positive and Negative Poisson Values

In the previous section, the most common composite materials such as fiberglass/epoxy or graphite/epoxy have been considered. Such composites have a stiffness ratio $E_{1}/E_{2}=8$–16. In particular, the material considered in the previous analyses is characterized by $E_{1}/E_{2}\approx 13$ (see Table 1). However, there is another class of highly orthotropic composite materials which are defined by $E_{1}/E_{2}=5000$–50,000 while using RP6410 or RP6442 as a polyurethane matrix, depending on the volume fraction of the fibers. Mechanical properties of these two constituents are listed in Table 2. The equivalent orthotropic coefficients for the present laminates are given in Table 3 which shows a stiffness ratio of approximately 42,800 and 10,530 respectively. Peculiarities and features of these laminates applied to flat plates have been described by Peel [64].

**Figure 15:**
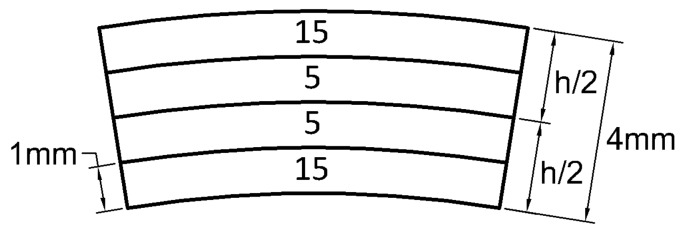
The (15/5)${}_{s}$ laminate.

This high stiffness ratio allows having a laminate with a high positive or negative engineering Poisson ratio of $\nu_{xy}$. A high Poisson is given by a balanced lamination and a negative Poisson is given by a symmetrical scheme. According to the studies by Peel [64], unbalanced lamination schemes ${\left[\theta /\alpha \right]}_{s}$ have very small (highly negative) Poisson ratios. In particular, the laminate ${\left(15/5\right)}_{s}$ (represented in Figure 15) has a Poisson ratio of about −34 for the Graphite/RP6410 material. Its engineering constants are listed in Table 4 alongside with those defined by the CFRP material.

**Figure 16:**
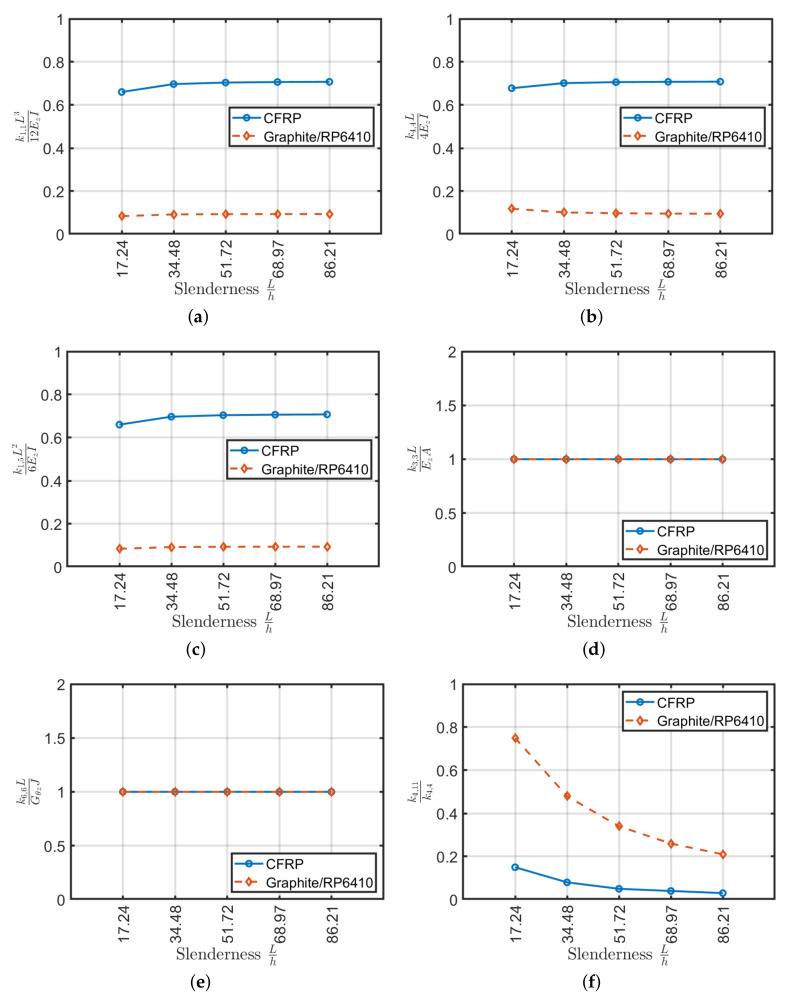
Stiffness coefficients as a function of slenderness for ${\left(15/5\right)}_{s}$ (**a**–**e**) and stiffness ratio $k_{4,11}/k_{4,4}$ (**f**).

The stiffness coefficients are carried out below for the materials listed in Table 4. The comparison between the two types of material is addressed on the same tubular profile of the previous cases. The coefficients of the stiffness matrix are evaluated and their variations with respect to beam slenderness are show in Figure 16. Both configurations have a small variation with respect to beam slenderness. The main difference is observed in the values of the stiffness coefficients $k_{1,1}$, $k_{4,4}$ and $k_{1,5}$ that compared to the same stiffnesses calculated by Euler–Bernoulli theory using homogenization a difference of approximately 90% in the case of Graphite/RP6410 and approximately 30% in the case of CFRP occurs. Regarding the coupling coefficients, both materials have significant values due to the unbalanced lamination scheme. In particular, Graphite/RP6410 will have lower coupling values due to the poor resistance in the transverse direction of the material, but by observing Figure 16f, a higher effect than the $k_{4,11}$ given by CFRP.

**Figure 17:**
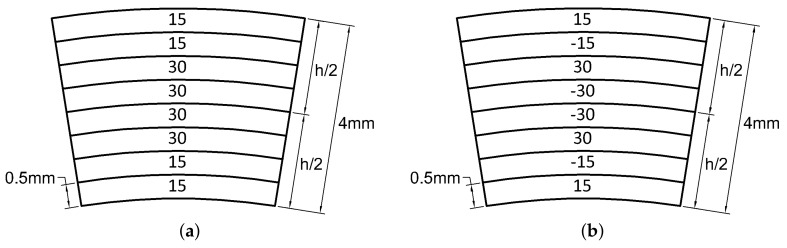
(**a**) The (15${}_{2}$/30${}_{2}$)${}_{s}$ laminate, (**b**) (±15/±30)${}_{s}$ laminate.

In the following, four configurations investigated numerically and experimentally by Peel [64] are used for studying the stiffness of tubular profiles. Stiffness ratios and equivalent Poisson ratios are listed in Table 5. The sequences are ${\left(15_{2}/30_{2}\right)}_{s}$ with negative Poisson ratio (NP1 and NP2) and (±15/±30)${}_{s}$ with a high Poisson ratio (HP1 and HP2). The enumeration 1 and 2 identifies the material used Graphite/RP6410 and Graphite/RP6442, respectively. The same nomenclature is used in Figure 17. The analysis was carried out by dividing the combinations according to the lamination schemes and comparing the two materials.

**Figure 18:**
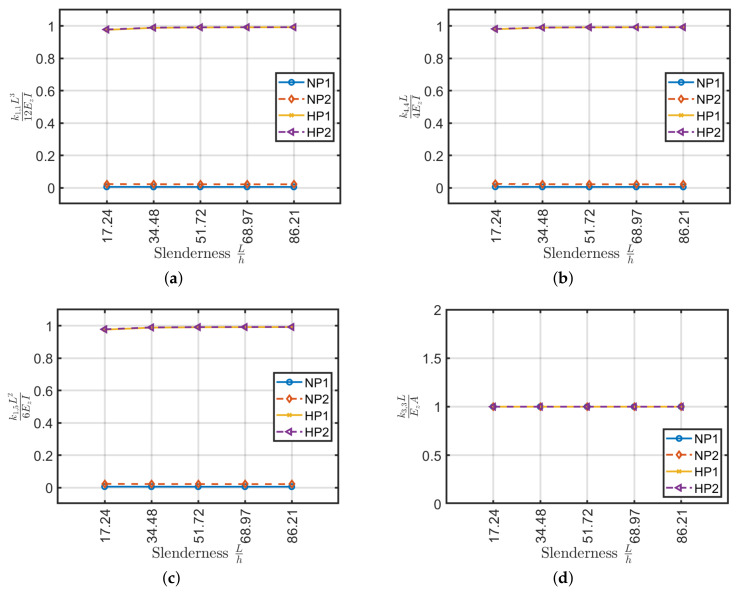

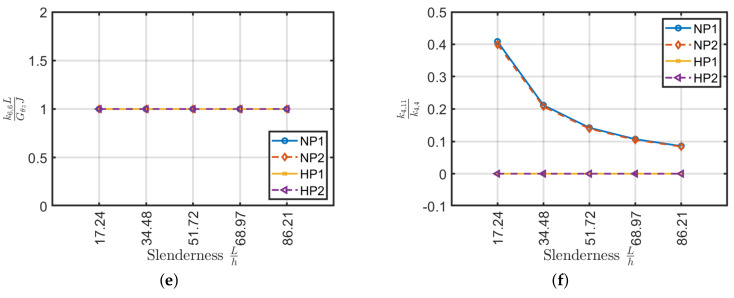
Stiffness coefﬁcients as a function of slenderness for (152/302)s and (± 15/± 30)s (**a**–**e**) and stiffness ratio k4,11/k4,4 (**f**).

The laminate ${\left(15_{2}/30_{2}\right)}_{s}$ (NP1 and NP2) and (±15/±30)${}_{s}$ (HP1 and HP2) are presented in Figure 18. The results for ${\left(15_{2}/30_{2}\right)}_{s}$ obtained were expected also considering the same given in Figure 16 where a smaller Poisson ratio was considered. The error given for the shear and bending behavior is approximately 98% on the contrary traction and torsion work well as in all the previous cases. Regarding the influence of the coupling coefficient $k_{4,11}$ with respect to the bending stiffness $k_{4,4}$, both materials behave in the same way, that is, tends to decrease by increasing beam slenderness. Such ratio goes from a value of about 40% (L/h= 12.24) to 10% (L/h=86.21) so it cannot be neglected.

A further comparison can be made with the previous lamination scheme ${\left(15/5\right)}_{s}$ presented in the Figure 16, which, observing the Graphite/RP6410, is characterized by a minor error towards the calculation of the stiffness coefficients with the Euler–Bernoulli formulation, but by a much greater influence of the coupling stiffness $k_{4,11}$, by about double.

The laminate ${\left(\pm 15/\pm 30\right)}_{s}$ (HP1 and HP2) is made up of highly orthotropic composite materials and a balanced lamination scheme. Assessment is depicted in the Figure 18. Compared to the previous laminate with a negative Poisson ratio, the balanced laminates generate stiffness values that are close to those obtained with the Euler–Bernoulli formulation for an isotropic material. This is expected by any balanced laminate, even in the case of highly orthotropic composite materials. The values of the coupling stiffnesses are negligible as a matter of fact $k_{4,11}/k_{4,4}$ is almost zero.

## 7. Validity and Limitations

After investigating several lamination schemes with standard and high stiffness ratios ($E_{1}/E_{2}$) according to beam slenderness and by comparing it to an equivalent homogenized isotropic Euler–Bernoulli beam model, the following remarks can be reported:

- cross-ply laminates, except laminate (90) have lower values, approximately 10% for L=1 m and highly variable as a function of slenderness with respect to the equivalent orthotropic model;
- (45) scheme has lower values than the reference of about 30% and invariant with respect to beam slenderness;
- (30) and (±30/30) scheme have lower values than the reference of about 55% and 5%, respectively, and they are invariant with respect to beam slenderness;
- unbalanced laminates with a fiber pitch angle of $\Delta \theta =5^{\circ }$, L.1 and L.2 have values lower than the reference of approximately 30% and 25%, respectively;
- ${\left(15/5\right)}_{s}$ scheme has lower values than the reference of approximately 30% for the CFRP configuration and 90% for the Graphite/RP6410;
- ${\left(15_{2}/30_{2}\right)}_{s}$ scheme has values very large errors of about 100% and it is invariant with respect to beam slenderness.

If cross-ply laminates are not considered, it is possible to observe that the calculation of the coefficients of the stiffness matrix is possible by using isotropic definitions of the Euler–Bernoulli theory (Equation (3) for balanced laminates and for angle-ply (±α/…) ones. It is remarked that angle-ply must have more than one ply in the stack, because as they increase the number of plies they tend to behave approximately as balanced with negligible coupling stiffness values.

The problem arises when cross-ply are also considered, because for low slenderness values, although balanced, they have stiffness values that deviate of about 10% from those defined from Euler–Bernoulli’s theory.

In order to analyze the couplings which might occur in the present laminates, the ABAQUS shell model is considered as a reference. Composite plate/shell Mindlin theory considers at the constitutive level the matrices [A], [B] and [D] which represent the membrane, the bending-membrane coupling, and the bending stiffness matrices, respectively.

It is well-known that the membrane-bending stiffness matrix ([B]) is zero for symmetric laminates and it is not zero for antisymmetric cross-ply (in particular $B_{11}=-B_{22}$ applies) and angle-ply (in particular $B_{16}\neq B_{26}\neq 0$ applies) configurations. Since both symmetric and antisymmetric schemes present errors with respect to the reference configuration, it is clear that such behavior is not due to only the membrane-bending coupling matrix [B]. In other words, balanced laminates have $A_{1,6}=A_{2,6}=0$ as well as cross-ply, however the latter presents larger errors in the definitions of $k_{1,1}$, $k_{4,4}$ and $k_{1,5}$ with respect to the same computed with balanced laminates.

**Figure 19:**
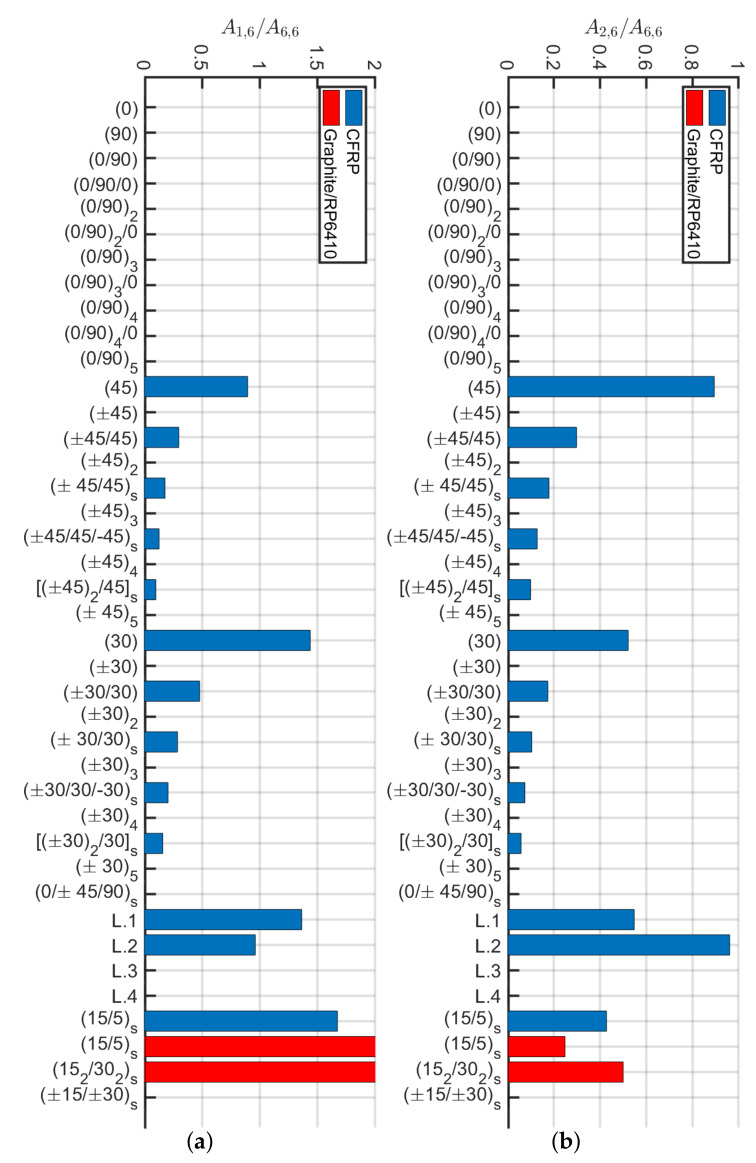
Ratios (**a**) $A_{1,6}/A_{6,6}$ and (**b**) $A_{2,6}/A_{6,6}$ for different laminates.

**Figure 20:**
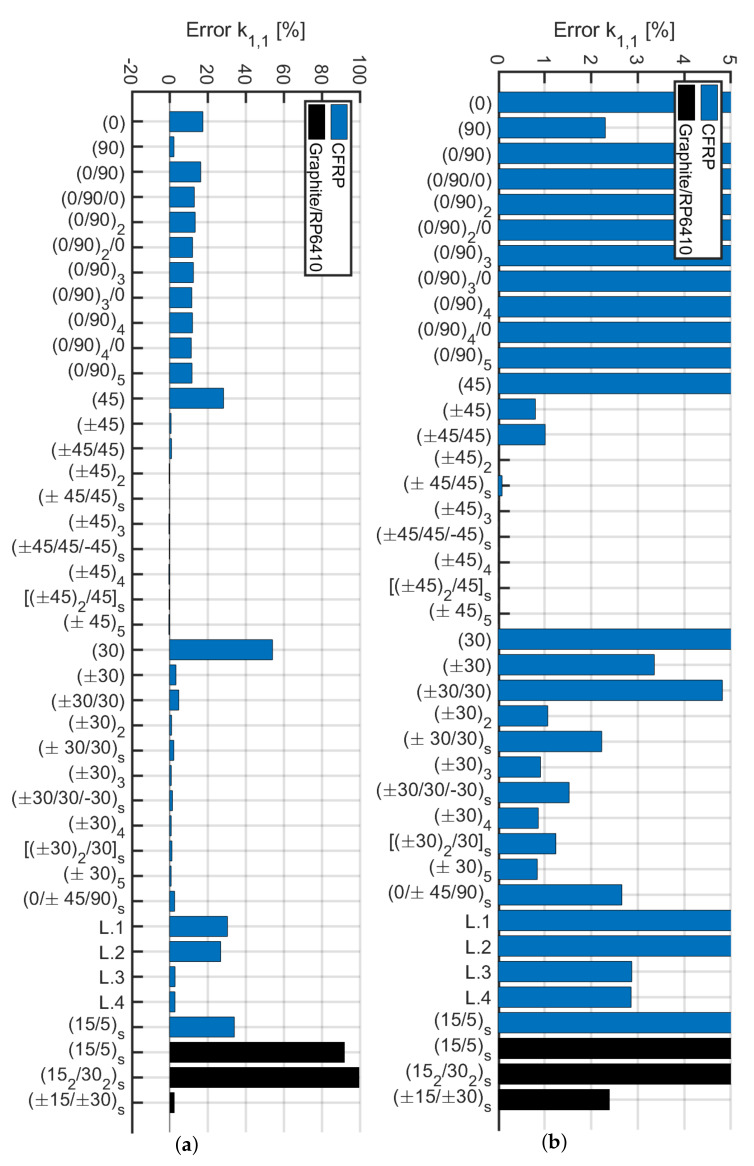
Percentage of error computing $k_{1,1}$ with the present methodology: (**a**) overall error; (**b**) the plot limited up to 5% of error.

A condition that allows using the analytical model of Sun et al. [40] and subsequently calculate the stiffness of the various beams via the Euler–Bernoulli theory for isotropic materials is sought. The authors investigated the following stiffness ratios $A_{1,6}/A_{6,6}$$A_{2,6}/A_{6,6}$, $A_{1,6}/A_{1,1}$, $A_{1,6}/A_{2,2}$, $D_{1,6}/D_{6,6}$,$D_{2,6}/D_{6,6}$, $D_{1,6}/D_{1,1}$, $D_{1,6}/D_{2,2}$. Differences among laminates and their configurations are mainly given by the presence of normal-shear couplings $A_{ij}$ and $D_{ij}$ when ij=16 or 26. In particular, it is relevant to analyze the ratio between the latter with the main stiffness terms when ij=11, 22 or 66. For the sake of conciseness, not all various combinations are reported, because only a few results are important to the present problem. Among the aforementioned list of ratios the only ones that result to be relevant were $A_{1,6}/A_{6,6}$ and $A_{2,6}/A_{6,6}$ (depicted in Figure 19). Such ratios are considered when the trend was similar to the correspondent error in the stiffness matrix components. To this aim, the variation of the error for $k_{1,1}$ has been taken as a reference for a beam of L=1 m and reported in Figure 20. Extensive details and plots regarding such ratios can be found in [62].

Among the ratios considered, leaving aside the cross-ply laminates, the one that has a trend similar to the error of $k_{1,1}$ is $A_{1,6}/A_{6,6}$. This is why a deeper investigation is provided to determine the validity of the stiffness calculation $k_{1,1}$ with the Euler–Bernoulli theory. Therefore, angle-ply laminates with lamination scheme of the type (α/−α/α) with the orientation of the laminae α defined with a difference of $2.5^{\circ }$ between plies are considered. In this way, it would be possible to determine the minimum orientation angle of the laminates for which the calculation of the stiffness with the Euler–Bernoulli formulas for an isotropic material is correct. The analysis was carried out by analyzing the stiffness coefficients ${\bar{Q}}_{ij}$ which represent the variables of $A_{ij}$ since the thickness of each ply is assumed constant.

(5) $$\frac{A_{1,6}}{A_{6,6}}=\frac{\sum_{k=1}^{3}\int_{\zeta_{k}}^{\zeta_{k+1}}{\bar{Q}}_{1,6}^{\left(k\right)}d\zeta }{\sum_{k=1}^{3}\int_{\zeta_{k}}^{\zeta_{k+1}}{\bar{Q}}_{6,6}^{\left(k\right)}d\zeta }=\frac{2{\bar{Q}}_{1,6}^{\left(\alpha \right)}\frac{1}{3}-{\bar{Q}}_{1,6}^{\left(-\alpha \right)}\frac{1}{3}}{3{\bar{Q}}_{6,6}^{\left(\pm \alpha \right)}\frac{1}{3}}=\frac{{\bar{Q}}_{1,6}}{3{\bar{Q}}_{6,6}}$$

**Figure 21:**
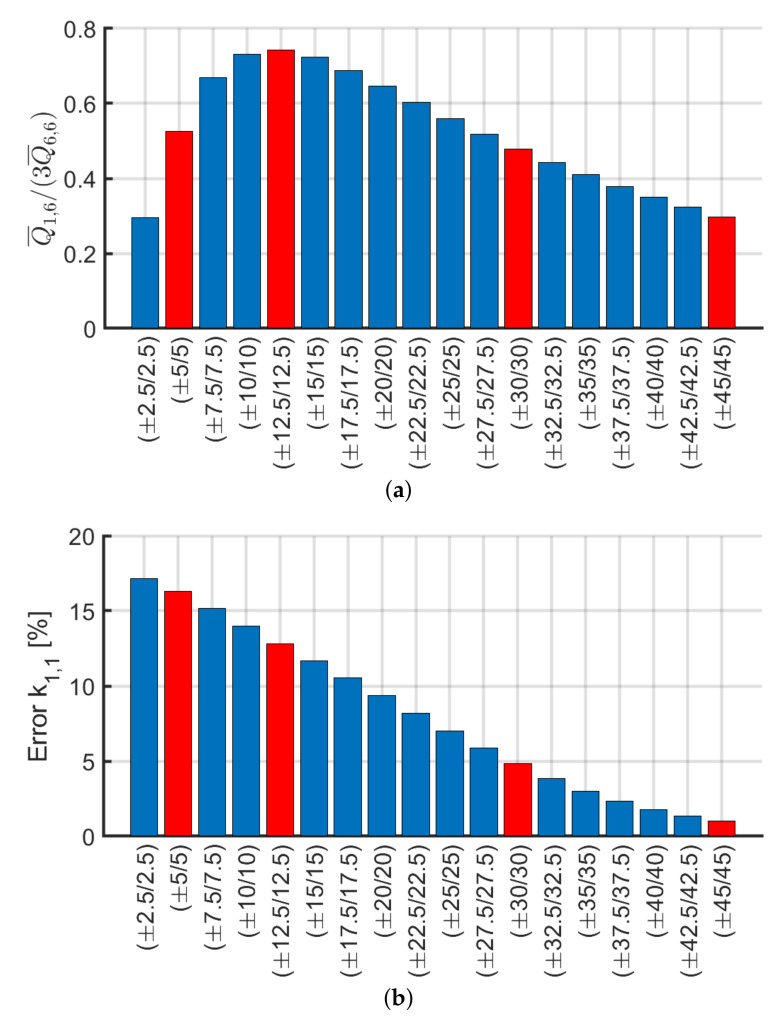
Trend by increasing α for laminates (α/−α/α) of: (**a**) ${\bar{Q}}_{1,6}/\left(3{\bar{Q}}_{6,6}\right)$; (**b**) error on $k_{1,1}$.

The lamination schemes analyzed and the values obtained are represented in Figure 21a. It is expected to find a trend for $k_{1,1}$ according to the value depicted in Figure 21a. In other words, the laminate (±5/5) should present an error in the calculation of the stiffness with the Euler–Bernoulli theory similar or slightly greater than the laminate (±30/30). On the contrary, Figure 21b shows that error on $k_{1,1}$ decreases by increasing α. It can be concluded that the error committed while stiffness is calculated using the Euler–Bernoulli theory is not a function of the ratio ${\bar{Q}}_{1,6}/\left(3{\bar{Q}}_{6,6}\right)$. Moreover, such a problem might be due to not uniform torsional and cross-section distortion effects which cannot be evaluated with the present model and must be verified via 3D modeling or higher-order beam formulation. However, the present methodology can be accurately utilized for angle-ply configurations when $\alpha >30^{\circ }$.

## 8. Conclusions

In conclusion, it has been verified by increasing the number of plies in cross- and angle-ply configurations that hollow circular beam behavior does not tend to be like the one of an orthotropic model (0).

Subsequently, an analytical method is proposed for the calculation of the stiffness matrix components $k_{i,j}$ for i,j=1,2,…,12 of an equivalent 3D Euler–Bernoulli beam model. The present method resulted to be a valid alternative for investigating composite hollow beams in a simplified manner without the need for complex 3D shell modeling. The methodology demonstrated to work well for balanced laminates with $A_{1,6}/A_{6,6}<50\%$ which corresponds to an error <5% on the shear and bending stiffness components. By accurately analyzing angle-ply (±α/α) configurations it has been demonstrated that a condition on the ply angle is $\alpha >12.5^{\circ }$ in order to have acceptable results.

On the contrary, the condition $A_{1,6}/A_{6,6}<50\%$ is not valid for cross-ply, that have $A_{1,6}=0$ and they present an error around 15% for small slenderness. These errors might be due to some non-uniform cross-section torsional effects that cannot be predicted with the present modeling.

A balanced bio-inspired Bouligand-type laminate has been observed to be a good candidate for the present approach because other than being completely defined by the present analytical procedure such scheme demonstrated to have high ductility, dissipated energy, and high load-carrying capability.

The present method is valid also for investigating hollow beams with orthotropic materials with high stiffness ratio $E_{1}/E_{2}$ that have a high Poisson ratio, which corresponds to a balanced laminate. On the contrary, symmetric laminates with the same materials that have a negative Poisson ratio cannot be modeled accurately (because their lamination scheme is not balanced).

## Figures and Tables

**Table 1:** Geometric and mechanical properties.

Geometric		Mechanical	
Average radius	27 mm	$E_{1}$	145,849.69 MPa
Length	1000 mm	$E_{2}$	11,030 MPa
Laminate thickness	4 mm (Fixed)	$\nu_{12}$	0.28
Layer thickness	Variable	$G_{12}$	6209.89 MPa
Area	678.58 mm${}^{2}$	$G_{13}$	6209.89 MPa
Polar inertia	497,400 mm${}^{4}$	$G_{23}$	3860.5 MPa
Inertia	248,774.1516 mm${}^{4}$

**Table 2:** Mechanical properties of laminates with high stiffness ratio [64].

Materials	*E* [MPa]	ϵ [%]
RP6410	1.65	3.30
RP6442	7.00	5.25
Graphite	276,000	-

**Table 3:** Mechanical properties of: Graphite/RP6410 and Graphite/RP6442 [64].

	$E_{1}$ [MPa]	$E_{2}$ [MPa]	$\nu_{12}=\nu_{13}$	$\nu_{23}$	$G_{12}=G_{13}=G_{23}$ [MPa]
Graphite/RP6410	120,000	2.85	0.41	0.502	0.949
Graphite/RP6442	126,600	12.02	0.41	0.504	4

**Table 4:** Engineering constants for ${\left(15/5\right)}_{s}$ using material from Table 2 and CFRP using material from Table 1.

Graphite/RP6410		CFRP	
$E_{x}$	4498.23 MPa	$E_{x}$	94,509.78 MPa
$E_{y}$	3.02 MPa	$E_{y}$	11,234.40 MPa
$\nu_{xy}$	−34.04	$\nu_{xy}$	0.342
$\nu_{yx}$	−0.023	$\nu_{yx}$	0.041
$G_{xy}$	23.33 MPa	$G_{xy}$	7395.17 MPa

**Table 5:** Stacking sequencies presented by Peel [64] stiffness and equivalent Poisson ratios.

Laminates	Material	$E_{1}/E_{2}$	$\nu_{\mathrm{xy}}$
NP1 ${\left(15_{2}/30_{2}\right)}_{s}$	Graphite/RP6410	42,800	−6.38
HP1 ${\left(\pm 15/\pm 30\right)}_{s}$	Graphite/RP6410	42,800	3.73
NP2 ${\left(15_{2}/30_{2}\right)}_{s}$	Graphite/RP6442	10,530	−6.15
HP2 ${\left(\pm 15/\pm 30\right)}_{s}$	Graphite/RP6442	10,530	3.72
